# Cognitive and Physical Activity-Related Aspects of Children Associated to the Performance of the Crunning Movement

**DOI:** 10.3390/jfmk6010009

**Published:** 2021-01-17

**Authors:** Ewan Thomas, Marianna Alesi, Garden Tabacchi, Carlos Marques da Silva, David J. Sturm, Fatma Neşe Şahin, Özkan Güler, Manuel Gómez-López, Simona Pajaujiene, Michele Basile, Ante Rada, Antonio Palma, Antonino Bianco

**Affiliations:** 1 Sport and Exercise Sciences Research Unit, Department of Psychology, Educational Science and Human Movement, University of Palermo, 90146 Palermo, Italy; marianna.alesi@unipa.it (M.A.); tabacchi.garden@libero.it (G.T.); antonio.palma@unipa.it (A.P.); antonino.bianco@unipa.it (A.B.); 2 CIEQV-Life Quality Research Centre, Escola Superior de Desporto de Rio Maior-IPSANTAREM, Avenida Dr. Mário Soares, 20413 RIO Maior, Portugal; csilva@esdrm.ipsantarem.pt; 3 Department of Sport and Health Sciences, Technical University of Munich, Uptown Munich Campus D, Georg-Brauchle-Ring 60/62, 80992 Munich, Germany; david.sturm@tum.de; 4 Department of Sport and Health, Faculty of Sport Sciences, Ankara University, Golbaşı Yerleşkesi Spor Bilimleri Fakültesi, Golbaşı, 06830 Ankara, Turkey; nesesahin@ankara.edu.tr (F.N.Ş.); oguler@ankara.edu.tr (Ö.G.); 5 Department of Physical Activity and Sport, Faculty of Sports Sciences, University of Murcia, Calle Argentina, s/n., 30720 Murcia, Spain; mgomezlop@um.es; 6 Department of Coaching Science, Lithuanian Sports University, Sporto 6, LT-44221 Kaunas, Lithuania; simona.pajaujiene@lsu.lt; 7 University of Palermo Sport Center (CUS Palermo), Via Altofonte, 80, 90129 Palermo, Italy; miter702@libero.it; 8 Faculty of Kinesiology, University of Split, Teslina 6, 21000 Split, Croatia; arada@kifst.hr

**Keywords:** crunning, socio-demographic, cognitive, fitness-tests

## Abstract

The aim of this investigation was to identify possible related factors associated to the performance of the crunning test in European children and adolescents. A total number of 559 children and adolescents (age range 6–14 years) of which 308 boys (55.1%) and 251 girls (44.9%), from seven European countries, were screened. A questionnaire concerning demographic and personal life-related factors and a cognitive assessment were performed. A regression analysis was conducted with the performance measures of the crunning movement. T-tests and ANCOVA were used to analyze sub-group differences. Boys have greater crunning performance values compared to girls (5.55 s vs. 7.06 s, *p* < 0.001) and older children perform better than younger ones (R2 −0.23; *p* < 0.001). Children with healthy and active habits (exercising or spending time with family members vs. reading or surfing the internet) performed better in the test. Children engaged in team sports had better crunning performances compared to those engaged in individual sports (6.01 s vs. 6.66 s, *p* = 0.0166). No significant association was found regarding cognitive-related aspects in either children engaged in team or individual sports and the crunning performance. Older and male children performed better in the crunning test than younger and female children. Physical activity-related aspects of children’s life are associated with crunning movement performance. No association was found between higher cognitive performance and the crunning test results.

## 1. Introduction

Physical fitness (PF) during childhood and adolescence has been deeply investigated [1,2,3]. It has been recognized that PF is an important health-related factor, which may predict health status in adulthood, and which may also help physical and cognitive development [1,4,5,6]. Inverse associations have been found between PF and cardiovascular disease, metabolic risk factors and adiposity, which overall suggest positive health-related aspects [5,7,8]. A recent review by Donnelly et al. [4], investigating the effects of PF on cognition, learning and brain structure and function in children aged 6–13 years old, found a general positive association regarding cognition and brain structure and function. However, the study failed to identify a specific set of activity parameters which may better promote the abovementioned aspects. However, despite different evidence existing regarding a positive association between physical activity (PA) and academic achievements [9], there are still inconsistencies regarding aspects of PA and cognitive function [4].

In order to promote the level of PF through PA in adulthood, it is important to adequately modify lifestyle and behaviours in childhood and adolescence [10,11]. Different investigations have suggested that children and adolescents who did not regularly practice PA are more prone to develop unhealthy habits as smoking or drinking alcohol [12,13], which would inevitably decrease PF. In addition, such aspects have also been linked to the familial socio-economic status, parental education and food habits [12,13]. Therefore, since lifestyle and familial education are important factors which influence both physical and psychological development of children and adolescence, these may be considered as important predictors of PF and health outcomes [14].

In order to quantify the levels of PF, a large body of scientific evidence exists which takes into account specific age ranges and genders [1,15,16,17,18], demographic characteristics [19,20,21] or more specific sub-populations [22,23]. Each investigation has evaluated different aspects related to PF, such as aerobic activity, strength, power, speed, coordination, flexibility, agility and other abilities, including for each of these, one or more field or laboratory tests to appropriately discriminate such abilities.

Despite the large number of fitness tests and physical evaluation batteries available, performance values or predictors of the crunning movement, a specific type of locomotion which combines running and crawling, are scarce. Patrick et al. [24] evaluated the inter-limb coordination during crawling in infants and adults, suggesting that a great involvement of coordination by the central nervous systems is required for quadrupedal locomotion. Other two investigations have included specific crawling exercises, within a fitness battery, to improve PF in a military environment [25,26]. The results of the studies underline that the military personnel increased the efficacy of different abilities, such as coordination, agility, speed, and power, following such interventions. Another investigation tried to determine a link between the Illinois agility test, which evaluates agility, and the crunning test. The results indicate a moderate correlation (r = 0.45) between the two tests [27], suggesting that a certain amount of agility is required to perform the crunning movement. In an attempt to understand if the crunning locomotion could provide further information than those offered from already known fitness tests, we included the crunning test in a previously discussed project [15]. This project, namely, the Enriched Sport Activity (ESA) program, was a physical activity intervention with the aim to improve fitness of children and adolescents across Europe by including cognitively enriched stimuli within specific warm-ups, prior to a structured physical activity [15]. However, such tests, together with the Leger shuttle run for aerobic assessment [28], were the only tests not influenced by the ESA intervention.

In order to clarify the characteristics of the crunning movement, this investigation will aim to identify lifestyle, physical activity and cognitive aspects associated with the results of this particular type of locomotion.

## 2. Materials and Methods

### 2.1. Participants

The sample was composed of 589 children of ages ranging from 6 to 14 years (aged 10.25 ± 1.76 years and 9.98 ± 1.87 years) of which 308 boys (55.1%, age 9.1 ± 1.3 years; weight, 34.9 ± 9.5 kg; height, 139.0 ± 10.4 cm) and 251 girls (44.9%, 10.2 ± 1.8 years; 39.4 ± 11.1 kg; 144.8 ± 14.3 cm), from 7 European countries (Italy, *n* = 164 of which 92 boys and 72 girls; Germany, *n* = 64 of which 41 boys and 23 girls; Portugal, *n* = 111 boys; Spain, *n* = 37 of which 17 boys and 20 girls; Lithuania, *n* = 85 of which 53 boys and 32 girls; Croatia, *n* = 50 of which 25 boys and 25 girls; and Turkey, *n* = 78 girls) within the ESA Program, an evidence-based exercise program cofounded by the Erasmus + Program of the European Union (Key action: Sport-579661-EPP-1-2016-2-IT-SPO-SCP).

Criteria for including participants were the following: 1. Able to perform the required tests; 2. Absence of a diagnosis of intellectual disability, visual or neurological impairments; 3. Absence of a diagnosis of other neurodevelopmental disorders. All children were recruited within a school or sport center. Before the inclusion of the children in the ESA program, a parent or legal representative of each child signed a declaration of informed consent. The study was conducted in accordance with the Helsinki Declaration (Hong Kong revision, September 1989) and the European Union recommendations for Good Clinical Practice (document 111/3976/88, July 1990). The study was approved with permission from the Lithuanian Sports University’s Research Ethics Committee in Social Sciences with approval No 579661-EPP-1-2016-2-IT-SPO-SCP (2018-02-05).

### 2.2. Study Design

The ESA Program aimed to improve children’s motor skills and executive functions through sport activities enriched by cognitive stimuli to enhance inhibitory control, working memory and shifting.

Detailed description of the study design and the ESA Program can be found elsewhere, describing both the cognitive tests and their administration and motor components, as well as the full description of the crunning test and administration procedures within the project [15,29].

### 2.3. Intervention Procedure and Assessment Tools

Each ESA trainer underwent a training procedure before the start of the intervention, in order to adopt a standardized procedure. The procedure was shared across the participating countries. This can be also found on a dedicated YouTube channel (ESA Program). Therefore, all testing procedures were performed in the same way and order. At the beginning of the project, each participant underwent a cognitive examination [29] and a physical evaluation [15], which were repeated at the end of the intervention. Before the start of the ESA training program, each participant also underwent a psychological test battery (ESA PTB, a 32 item questionnaire which includes questions regarding personal information, lifestyle-related factors and school-associated information), which was administered on two different occasions prior to the inclusion of the participants in the ESA program. On the first day, the participants underwent a cognitive assessment which lasted around 1 h [29]. On the second day, a questionnaire was administered, which included questions related to habits, family, recreational and social aspects. We retrospectively evaluated the predicting factors of the crunning performance through these personal aspects.

The crunning performance, measured as the time in seconds needed to perform the test (10 m distance), was the dependent variable collected through the crunning test, included in the ESA fitness battery. This battery is composed of six fitness tests (standing broad jump, seated medicine ball throw, 20 m shuttle run test, 30 m sprint, Illinois test and the crunning test) to assess different skill-related components at once [30], but for the purpose of the present paper, only the outcomes of the crunning test have been considered [15]. Low values in the test measure indicate better performance.

For the purpose of this study, gender and age were treated as covariates, while a number of predictors were taken into account. These predictors included sport-related aspects, spare time-related aspects and cognitive or neuropsychological aspects.

Each aspect is discussed below.

(a) Sport-related aspects: Sport type (individual/team and exercise intensity) and sport frequency.

Individual/team sport type classification was manually distinguished by the authors on the basis of the sport practiced. The American Heart Association classification for sport was adopted [31] for grouping the activities in relation to cardiovascular function. These are divided by static and dynamic activities, and each is stratified according to exercise intensity, categorized as low, moderate or high. Current sport frequency and past sport frequency were measured in hours per week and relevant data were collected through a dedicated section of the ESA PTB.

(b) Spare time-related aspects (frequencies in h/week): Aspects regarding time spent with parents and siblings, reading, going to the cinema, theatre or museums, surfing the internet, playing with electronic games, going out with friends, going to the gym, going to the park or shopping were evaluated. All these items were collected through a dedicated section of the ESA PTB.

(c) Cognitive/neuropsychological variables: School marks (National language, Maths, Anthropology and History, Geography, Physical education, Foreign language), inhibitory control, working memory, shifting of attention. The ESA PTB was used to collect the school mark items. The other items’ assessment was performed through the neuropsychological tests derived by the Inquisit Lab platform (Inquisit 6 (for Windows version 6.1). 2020. Retrieved from https://www.millisecond.com): the Color Word Stroop (CWS) task for the inhibitory control, measured by the time needed to select the right color within the stroop test; the Digit Span Test (DST) for working memory, measured by the number of recalled digits in an exact order and in a reversed order; and the Trail Making Test (TMT) for shifting of attention, measured by error number and execution time. For further details regarding the cognitive test administration, please refer to Gentile et al. [29].

### 2.4. Statistical Analysis

Data are presented as means and standard deviations. The Student’s t-test was used to assess differences in the crunning test for each variable (i.e., gender, sport type). To estimate differences in the crunning performance by country, age range and sport type, the ANCOVA test was performed. Age and gender were used as covariates for all analysis.

Linear regression analyses were performed to evaluate correlations between the crunning test and sport frequency, spare time-related aspects, cognitive/neuropsychological aspects. Statistical significance was set for *p* < 0.05. All estimates were adjusted for gender and age. The software STATA/MP 12.1 (StataCorp. 2011. Stata Statistical Software: Release 12. College Station, TX, USA: StataCorp LP.) was used for the statistical analysis.

## 3. Results

### 3.1. Gender and Age

Mean time to perform the crunning test was 6.25 s (SD 2.416, *n.* = 589). Better crunning performances were observed for males compared to females (independently from age) and for older compared to younger children (independently from gender) (Table 1). The regression coefficient for age was −0.23 (SE 0.051, *p* = 0.000, *n* = 589).

### 3.2. Demographic Factors

Table 2 shows the differences in the crunning movement performances by demographic and SES factors. Similar characteristics across participants for each county were observed (Italy, 8.5 ± 1 years; 34.3 ± 9.8 kg; 135.0 ± 9.8 cm; boys, 8.4 ± 1 years; 33.9 ± 9.7 kg; 135.3 ± 9.7 cm and girls, 8.6 ± 1.1 years; 34.8 ± 10.1 kg; 134.7 ± 10 cm; Germany, 8.9 ± 0.8 years; 37.6 ± 9.9 kg; 137.8 ± 7.5 cm; boys, 9.0 ± 0.8 years; 37.6 ± 8.4 kg; 138.6 ± 7.3 cm and girls, 8.7 ± 0.7 years; 37.4 ± 12.7 kg; 136.3 ± 7.9 cm; Portugal, boys, 10.9 ± 1.7 years; 139.1 ± 15.3 cm; 39.4 ± 9.3 kg; Spain, 10.5 ± 1.0 years; 142.7 ± 20.4 cm; 45.1 ± 23.1 kg; boys, 10.4 ± 0.9 years; 145.8 ± 14.1 cm; 43.2 ± 12.4 kg and girls, 10.4 ± 1.0 years; 144.7 ± 13.2 cm; 40.2 ± 10.4 kg; Lithuania, 9.9 ± 1.13 years; 35.7 ± 8.0 kg; 144.0 ± 9.0 cm, boys 9.9 ± 1.19 years; 36.4 ± 8.9 kg; 144.0 ± 9.0 cm and girls 10.1 ± 1.02 years; 34.6 ± 5.8 kg; 142.0 ± 11.0 cm; Croatia 9.4 ± 0.5 years; 35.0 ± 8.2 kg; 138.3 ± 7.6 cm; boys, 9.7 ± 0.5 years; 36.5 ± 7.6 kg; 140.7 ± 7.4 cm and girls, 9.3 ± 0.5 years; 34.7 ± 8.3 kg; 137.8 ± 7.6 cm; and Turkey, girls, 10.8 ± 1.8 years; 134.9 ± 12.7 cm; 45.3 ± 10.9 kg). Significant differences in the crunning movement were found across countries.

### 3.3. Sport-Related Aspects

Among the sport-related aspects, the following were found to be associated to better performances: team sport type, with children practicing team sports performing better than those practicing individual sports (h/week) (Table 3).

No differences were found across different sports, current sport frequency, sport type (based on the American Heart Association classification (impact)).

### 3.4. Spare Time-Related Aspects

Regarding spare time-related aspects, the following items were correlated to the crunning performances. In general, physical activities such as attending a gym, going to the park or going out with friends were positively associated to performance outcomes (Table 4).

Furthermore, spending more time with parents and siblings seems to be associated with better performances.

### 3.5. Cognitive/Neuropsychological Aspects

In general, very low regression coefficients were found. Children with lower school marks had better crunning performances, such relation was present in either children practicing teams and individual sports (Table 5). No significant correlations were found between all the inhibitory control items and the crunning movement for children practicing team or individual sports, with higher times needed to select congruent, incongruent and control trials associated to lower times needed to perform the test. Working memory items were not correlated to the crunning performance.

Items related to the shifting of attention showed positive but very low correlations to crunning times (with higher number of errors and higher time to perform the test correlated to lower crunning performances), with the only exception of the time to recognize numbers and letters in children engaged in team sports, which revealed significant correlation but very low coefficients.

## 4. Discussion

With this investigation, we aimed to determine possible factors associated with the performance of the crunning movement. As expected, in our population of European children, males performed better compared to females and older children performed better than younger ones.

Such aspects have been reported by various authors across different motor domains. Tomkinson et al. [1] reported for a sample of over 2 million children performances, increases across age groups from 9 to 17 years old, with males performing better than females in terms of strength, power, agility and aerobic capacity. However, the results are in favour of females regarding balance, flexibility and coordination. Similar results are reported by another investigation in a sample of 3804 children ranging between 6 to 10 years of age. Again, boys perform better than girls regarding strength, power, speed, agility and aerobic capacity but not flexibility, with performance increases according to age [32]. Both aspects are consistent with growth and physical maturation [33,34].

### 4.1. Demographic Aspects

The results we obtained regarding the crunning movement performance show differences related to each country with better performances from the Portuguese children and worst performances from the Turkish children. It has to be noted that the Portuguese sample was composed only of male children, while the Turkish sample was composed only of female children. Such results are consistent with the general outcomes provided above. Furthermore, it is not uncommon to appreciate geographical differences when considering different populations of same age ranges, for example, regarding speed in the 20 m sprint between 6-year-old Greek (5.05 ± 1 s) [35] and Lithuanian boys (5.8 ± 1.2 s) [36], or for jumping performance of 10-year-old Colombian girls [20] (110.2 cm), Australian girls (103.25 cm) [37] or South African girls (149.3 cm) [38] in the standing long jump. Differences are also present between the same country when considering rural and city areas [39]. The authors of this latter study indicated that these differences between areas possibly influence individual habits and therefore the level of physical activity which each children experiences. A review by Carlin et al. [40] also analysed the association of environmental factors and levels of PA. The study associated increased levels of PA in neighbourhoods which provided facilities, parks and public equipment, and found the opposite for those with fewer facilities. Therefore, environmental and cultural aspects may possibly explain the difference reported between the test performance of each country within the present investigation.

### 4.2. Sport and Spare Time-Related Aspects

In the previous sections, the influence of habits regarding the activity levels was evaluated [41]. The results we obtained in this sample of European children confirm that positive habits and lifestyles are associated with improved performance of the crunning movement. For instance, the better results obtained in those children engaged in gym activities or those going out to the park in their spare time. Our results are in line with a systematic review analysing the relationship between outdoor time and PF in children [42]. The authors report an overall positive effect of outdoor time on PF, however, with no specific effect on musculoskeletal fitness. Another cross-sectional investigation aimed to understand how sedentary behaviours or screen time could affect motor skills in children aged 5–16 years [43]. Screen time in particular was associated with lower physical activity, with greater effects on adolescents compared to children and on girls compared to boys. This aspect, if linked to increased sedentary behaviours, could lead to increased fat mass and therefore decreased PF amongst children [44].

In addition, our results also show that children engaged in team sports performed better than those engaged in individual activities, independently of the activity intensity. Other investigations comparing individual and team sports have reported similar results, for example, Morano et al. [45] evaluated physical and psychological factors among children, finding that children engaged in team activities had better shuttle run results for aerobic performances compared to their peers who were engaged in individual sports, and were also less dissatisfied with their body image. Jukic et al. [46] examined the differences in fundamental motor skills in a sample of under-10 soccer players, indicating that greater motor skills were positively related to gross motor quotient and locomotion skills. School children’s enjoyment and cohesion during sport activities can predict physical improvements, and those engaged in team sports show significantly higher levels of enjoyment [47]. Being engaged in team sports has also been associated in children with increased motor skill proficiency [48], with greater associations in boys compared to girls. When neurobiological integrations are analysed, it is possible to see that boys involved in team sports have thinner cortices in the frontal lobe compared to those engaged in individual activities. This aspect indicates a faster maturation of the frontal cortex related to an advantage of frontal areas functioning [49]. These are factors that altogether are determinant to adequately perform a complex skill as the crunning movement, which requires the use of upper and lower limbs simultaneously.

### 4.3. Cognitive and Neuropsychological Aspects

Relative to cognitive and neuropsychological aspects, two main findings have been associated to the performance. Firstly, academic achievement is negatively linked to the performance results and, secondly, cognitive function is not linked to crunning performance neither for children practicing team sports nor for those practicing individual sports. Our findings are generally in contrast with other investigations. Emerging literature has positively linked cognitive performance and academic achievement to sport performance [50]. Such associations are also seen in intervention studies where both cognitive functioning and academic achievement are increased following a physical intervention [51,52,53]. However, the majority of studies have proposed aerobic activity as the main typology of physical activity included within their interventions [54]. These interventions are seen to act by improving brain structure and function [4]. However, within our study, neither a longitudinal intervention was undertaken nor the crunning movement involves aerobic metabolism activation. It is possible that the nature of the crunning movement itself requires a different cognitive demand compared to the cognitive and neuropsychological measures assessed.

Limitations of this study are the following: (1) The sample for each country was collected within one school, therefore, it was not possible to evaluate specific local aspects but only broad geographical differences. (2) Not all countries had the availability of both male and female participants. (3) The classification for sport intensity was based on broad characteristics of sporting activities (i.e., soccer, basket, gymnastics, etc.), therefore it is not possible to associate specific aspects related to different approaches between countries. (4) Regarding the cognitive and neuropsychological assessment, not all children and adolescents from the different countries were screened because they were not present at the time of the evaluation. Therefore, the sample which is available for providing information regarding results for such aspects is restricted. Indeed, a broader sample would be required for greater consistency of the test. There is a need to identify the sensitivity of the test to specific physical qualities.

Therefore, it will be important in future investigations to compare the present results to other populations in order to verify the applicability of the findings here provided.

The present study is the first known study to have evaluated and tried to identify variables associated to the crunning movement. Therefore, it will be necessary to consider gender and physical activity differences among individuals if planning to include the crunning test within a test battery.

## 5. Conclusions

The present investigation detected different factors associated with the performance of the crunning movement. These are related to lifestyle and cognitive factors which may influence performance of the crunning movement. These associated variables need to be considered when comparing the results of the crunning movement test, especially across populations. Special attention must be paid regarding gender and previously practiced physical activity. The specificity of the crunning test still needs to be understood within the context of a fitness evaluation.

## Figures and Tables

**Table 1:** Differences in the crunning test performances by gender and age range.

	n	Mean (s)	SD (s)	*p*-Value
Tot	589	6.25	2.416	
Gender				0.000 ^a^
male	318	5.55	2.306	
female	271	7.06	2.289	
Age range				0.000 ^b^
6–8	162	7.2	2.946	
9–11	298	5.99	1.97	
≥12	129	5.65	2.291	

SD: Standard deviation; Tot: Total number; ^a^ Estimated through paired Student’s *t*-test. ^b^ Estimated through ANCOVA. All estimates were adjusted for gender and age.

**Table 2:** Differences in crunning test performances by country and socio-economic status.

	n	Mean (s)	SD (s)	*p*-Value ^a^
Country	589	6.25	2.416	0.000
Croatia	50	5.26	1.325	
Germany	64	5.62	3.116	
Italy	164	7.69	2.501	
Lithuania	85	5.36	1.503	
Portugal	111	4.31	1.151	
Spain	37	6.37	0.508	
Turkey	78	8.03	1.709	

SD: Standard deviation; ^a^ Estimated through ANCOVA. All estimates were adjusted for gender and age.

**Table 3:** Differences in crunning test performances by sport-related aspects.

	n	Mean (s)	SD (s)	*p*-Value ^a^
Sport type	384	6.18	2.316	0.0166
Individual	104	6.64	2.321	
Team	280	6.01	2.294	
Sport type (*n* = 5 *)	384	6.18	2.316	0.1139
	n.	R^2^.	SE	*p*-value ^b^
Sport frequency (h/week)	467	0.14	0.101	0.168

SD: Standard deviation; SE: Standard error; ^a^ Estimated through paired Student’s *t*-test. ^b^ Estimated through linear regression analysis. * Five sport categories were included, according to the American Heart Association classification adopted.

**Table 4:** Differences in crunning test performances by spare time-related aspects. All estimates were adjusted for gender and age.

Activity (Times/Week)	n	R^2^	SE	*p*-Value ^a^
Going to the gym	531	−0.16	0.079	0.049
Going to the park	537	−0.29	0.094	0.002
Going out with friends	534	−0.25	0.078	0.002
Shopping	538	−0.06	0.097	0.53
Going to the cinema/theatre/museum	532	−0.1	0.128	0.447
Surfing the internet	538	−0.06	0.071	0.417
Playing electronic games	538	0.13	0.07	0.056
Reading	539	−0.05	0.075	0.535
Spending more time with parents and siblings	541	−0.17	0.087	0.049

SE: Standard error; ^a^ Estimated through linear regression analysis. All estimates were adjusted for gender and age.

**Table 5:** Associations between the crunning test performance and cognitive/neuropsychological aspects for team and individual sport practitioners.

	Team			Individual				
	n	R^2 a^	SE	*p*	n	R^2 a^	SE	*p*
School marks								
National language	205	0.47	1.58	0.000	75	0.15	0.40	0.012
Maths	70	0.29	2.49	0.000	77	0.15	0.33	0.009
Anthropology and History	65	0.19	2.49	0.000	73	0.11	0.31	0.041
Physical Activity	62	0.22	2.44	0.003	76	0.17	0.51	0.004
Foreign language	66	0.28	2.27	0.000	68	0.14	0.37	0.027
Inhibitory control (CWS)								
Congruent trial selection (s)	150	−0.59	9.03	0.845	91	−0.39	2.15	0.735
Incongruent trial selection (s)	150	0.69	2.17	0.182	91	0.55	2.21	0.116
Control trial selection (s)	150	0.39	2.18	0.004	91	0.50	2.59	0.254
Working memory (DST)								
Forward recall of numbers (n)	156	0.16	0.89	0.000	77	0.07	0.55	0.064
Forward recall of numbers before 2 consecutive errors (n)	156	0.20	1.38	0.000	77	0.08	0.55	0.060
Backward recall of numbers (n)	156	0.15	0.129	0.000	77	0.12	0.52	0.017
Backward recall of numbers before 2 consecutive errors (n)	156	0.15	1.19	0.000	77	0.06	0.53	0.126
Shifting of attention (TMT)								
Errors in numbers (n)	148	0.18	1.05	0.000	79	0.06	0.44	0.138
Time in numbers (s)	148	0.00	0.000	0.018	79	0.00	0.000	0.256
Errors in numbers and letters (n)	148	0.23	1.26	0.000	79	0.18	0.32	0.024
Time in numbers and letters (s)	148	−0.00	0.000	0.000	79	−0.00	0.000	0.118

SE: Standard error; ^a^ Estimated through linear regression analysis. All estimates were adjusted for gender and age.

## Data Availability

Data available on request due to restrictions eg privacy or ethical.
